# A bio-inspired approach for the synthesis of few-layer graphene using beetle defensive gland extract†

**DOI:** 10.1039/d3ra08733f

**Published:** 2024-02-16

**Authors:** A. P. Ajaykumar, K. Nikhila, Ovungal Sabira, Kodangattil Narayanan Jayaraj, Sudhir Rama Varma, V. A. Rasheed, V. S. Binitha, Kalapparambil Sreeja, Resmi M. Ramakrishnan, Annet Babu

**Affiliations:** a Division of Biomaterial Sciences, Department of Zoology, Sree Neelakanta Government Sanskrit College Pattambi Palakkad Kerala India ajaykumar@sngscollege.org; b Basic Sciences Department, Centre for Medical and Bio-allied Health Sciences Research, Ajman University Ajman United Arab Emirates j.narayanan@ajman.ac.ae; c Clinical Sciences Department, Centre for Medical and Bio-allied Health Sciences Research, Ajman University Ajman United Arab Emirates; d Department of Zoology, Sree Narayana College Nattika Thrissur Kerala India; e Department of Chemistry, Sree Neelakanta Government Sanskrit College Pattambi Palakkad Kerala India

## Abstract

Graphene exhibits remarkable properties and holds substantial promise for diverse applications. Its unique combination of thermal, chemical, physical, and biological properties makes it an appealing material for a wide range of uses. But, the lack of an economical and environmentally friendly approach to synthesize good-quality graphene represents an immense challenge for the scientific community. What makes this research unique is the utilization of the defensive gland extract from the beetle species *Luprops tristis* (Order: Coleoptera, Family: Tenebrionidae) to synthesize a few layers of graphene (FLG). This innovative incorporation of natural resources and exploration of biologically inspired methods provides an eco-friendly and cost-effective alternative to conventional graphene production techniques. The exfoliated graphene displayed antimicrobial effects against both Gram-positive (*Staphylococcus aureus*) and Gram-negative (*Escherichia coli*) bacteria, with particularly potent bactericidal activity. Additionally, the cytotoxicity assay demonstrated the anti-cancer properties of biosynthesized graphene against Daltons Lymphoma Acetic (DLA) cells.

## Abstract 2

Exfoliation of graphite.

## Graphical abstract

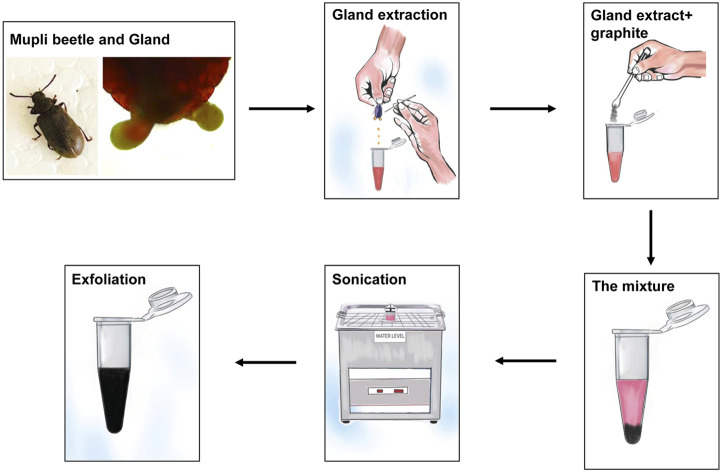

## 1. Introduction

Graphene is a newly emerged horizon in the world of science; it is a carbon allotrope that is currently highly in-demand in both research and commerce.^1^ Structurally, it's thought to be a mother material for other carbon allotropes, including fullerenes, graphite, charcoal and carbon nanotubes.^2–4^ The term “graphene” was first introduced by Boehm in 1986, for the single layer of carbons present in graphite.^5^ Owing to its superior thermal conductivity, mechanical strength, current density, electron mobility, and surface area, graphene has been a popular focus of research in the present decade and is being explored and used in a variety of fields, from electronics to biomedical applications.^6^ The extraordinary properties of graphene have garnered significant interest, leading to its recognition as a “magical material.” The structure of a single layer of graphene is composed of a web of sp^2^ carbon atoms, which are interconnected in two dimensions by covalent bonds with a bond length of 0.142 nm.^7^ In graphite, these graphene layers get laid on top of one another by van der Waals forces acting in between them.^8^ Most of the exfoliation processes aim to weaken these forces acting between the graphene layers and result in the graphite exfoliation. In 2004, Novoselov and Geim demonstrated graphene preparation from graphite by a scotch tape peeling method.^9^ It is revealed that about 99.99% of the electromagnetic radiations are strongly blocked by graphene.^10^ Electronics, biotechnology, and medical sectors, among others, can all benefit from using graphene's electrical properties.^3^ As a result, there is a lot of interest in the research on graphene synthesis all around the world.

Two distinct methods have been employed in the synthesis of graphene, known as the bottom-up and the top-down approaches.^6,11–14^ The former involves synthesizing graphene from carefully designed molecular building blocks that undergo chemical reactions to form covalently bonded 2D networks. On the other hand, the latter approach involves exfoliating graphite to form graphene.^2,15^ Top-down tactics can be implemented under various environmental variables.^6^ Besides the mechanical cleavage based on the scotch tape method, liquid-phase exfoliation (LPE) methods are gaining prominence because they are extremely versatile, potentially up-scalable, and can be used to deposit graphene in a variety of environments and on substrates that mechanical cleavage and growth methods cannot.^6^ Exfoliation is the process by which individual separation of carbon sheets to one or more tiny sheets of graphene takes place.^16^ Chemical or thermal approaches are the usually applied methods to exfoliate graphene. Graphene has been exfoliated using a variety of organic solvents, including *N*-methyl-pyrrolidone (NMP), dimethyl-formamide (DMF), dimethyl sulfoxide (DMSO), and ethylene glycol (EG).^17^ Their high boiling points are one disadvantage of these solvents.^18^ It is widely acknowledged that polyphenolic compounds function well as solvents for exfoliating graphite, generating graphene.^19–21^ However, some of these strategies end up in releasing toxic chemicals.^22,23^ Certain recent investigations have demonstrated environment friendly methods using natural reducing agents like; extracts from plant parts and aromatic compounds, carbohydrates *etc.*^24^ But most of these methods are based on plant origin and only a few are of animal origin.^25–27^ Microbial exfoliation remains the focal point of a great deal of these biological approaches. However, the microbial methods suffer from a major drawback; its intricate procedure for sustaining cell cultures as well as refining particular components makes it complicated.^24^

The experimental insect, *Luprops tristis* (Order: Coleoptera, Family: Tenebrionidae) 8 mm long, black coloured, plant detritus eating beetle, found in various parts of India. Following summer rain, these nocturnal beetles make massive invasions on buildings and residential areas, thus creating nuisance to people. This is a regular phenomenon commonly found throughout the rubber plantations of Kerala. The immature fall of tender leaves of rubber due to various seasonal diseases contribute to their food and this ensures their presence throughout the season.^28^ They are very special among other Coleopterans on having a period of oligo-pause (lasting up to 9 months) which is an intermediate between quiescence and diapause.^29–31^ Abdominal gland of Mupli beetle is known as pygidial glands which have two sets of secretary lobes, collecting canal and collecting reservoir. The yellow secretion of this gland is their defensive tool against predators. Due to their extensive distribution, the propensity to congregate in residential areas, lack of natural enemies, and unique ability to blend in with leaf debris, they are impossible to control using conventional methods. Even though they usually stay harmless to human kind, the defensive secretion oozed out of their abdomen when disturbed (like picked up, squeezed) causes severe skin burn.

Previous research carried out in our laboratory showed that 2,3-dimethyl-1,4-benzoquinone, 2,5-dimethylhydroquinone and 1,3-dihydroxy-2-methylbenzene are abundant in the defensive secretion of the Mupli beetle, *L. tristis*.^32^ Furthermore, we successfully synthesized metal nanoparticles using the extract derived from the defensive glands of *L. tristis*.^33^ Hence, the aim of this study is to create a unique, cost-effective, and environmentally friendly method for synthesizing few-layer graphene through liquid phase exfoliation of graphite, utilizing extracts from the defensive secretion of the Mupli beetle, *L. tristis*. The graphene produced through this biologically inspired approach demonstrated encouraging antimicrobial capabilities and cytotoxic effects against cancer cells.

## 2. Materials and methods

### 2.1. Extraction of defensive gland secretion

The insect, *Luprops tristis* (Order: Coleoptera; Family: Tenebrionidae) also referred to as Mupli beetle, is a dark coloured litter inhabiting one. A total of 200 beetles were collected (handpicked) from the Ladies Hostel of Sree Neelakanta Government Sanskrit College, Pattambi (10°8′ N 76°7′ E) in the Kerala state of India. The captured ones were subsequently taken to the lab in porous plastic jars (containing closures), making sure that the beetles had access to appropriate abiotic conditions. It was followed by the extraction of the defensive gland.

**Fig. 1:**
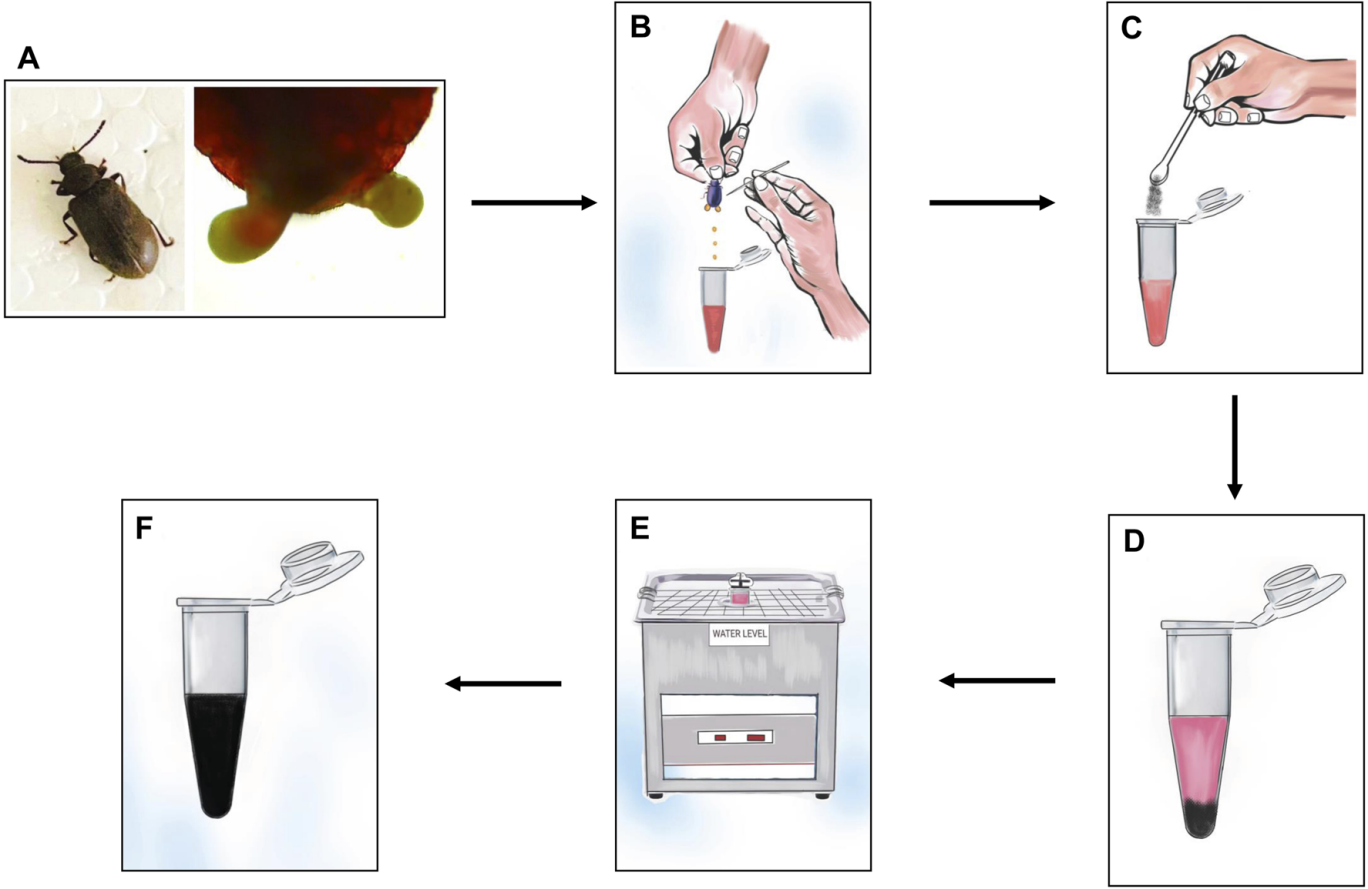
Diagrammatic representation of the exfoliation of graphite using the defensive gland extracts of the beetle *L. tristis*. (A) The Mupli beetle, *Luprops tristis* and the defensive gland of the beetle exposed from the body of the insect. (B) Extraction of defensive gland. (C) Adding powdered graphite flakes into the defensive gland extract. (D) The reaction mixture, which includes graphite flakes, is located at the bottom of the Eppendorf tube. (E) Sonication of the immiscible mixture for one hour. (F) Exfoliated graphene after sonication.

Defensive secretion was collected from both male and female beetles. The defensive glands (Fig. 1A) were found between the 7th and 8th sternum. To locate the defensive gland, the elytra and terga of the insects were gently placed sideways by placing them on the first and forefingers. The posterior part of the abdomen was then sterilized using cotton soaked in deionized water. The beetle was carefully disturbed with a fine needle, and pressure was applied to the abdomen to release the gland. A sharp needle was used to break the protruded gland, and care was taken to prevent any interference with faecal-like substances.^32^ The obtained extract was subsequently gathered in a 500 μl Eppendorf tube, already containing 300 μl of deionized water. This resulting mixture, consisting of 300 μl of deionized water and 200 μl of gland extract, was employed for the exfoliation of graphite.

### 2.2. Exfoliation of graphite dispersion

The defensive gland mediated graphite exfoliation was carried out by adding gland extract of 30 beetles in 300 μl of deionised water. The extract was then centrifuged in a micro centrifuge (1 ml capacity) at 10 000 rpm for 1 min. The reaction mixture was prepared by adding 600 μl of defensive gland extract (60 gland equivalents) and 400 μl (10 μg/400 μl) of powdered graphite flakes (Sigma Aldrich Chemicals India Pvt. Ltd) in deionised water. The immiscible mixture was subjected to ultrasonication using an ultrasound bath sonicator with 100 W ultrasonic power (LABMAN ULTRASONIC CLEANER-LMUC-3) for a duration of 1 hour at a frequency of 40 kHz. The entire exfoliation process is illustrated in Fig. 1(B–F). To avoid any temperature increase in the solution caused by energy generation during ultrasonication, the circulating water thermostat was set at 15 °C. The process was followed by 3 minutes of centrifugation at 10 000 rpm. Finally, the exfoliated graphene was separated and used for further analysis.

### 2.3. Characterization of exfoliated graphene

The morphology of exfoliated graphene was analysed on an HR-TEM instrument (Joel/JEM 2100) having a 0.14 nm point resolution at 200 kV. The thickness of the graphene sheet was analysed using atomic force microscope (Multimode 8-HR, containing Bruker's exclusive ScanAsyst). Raman spectroscopic analysis was carried out on a confocal Raman microscope with AFM imaging (WiTec alpha 300, Germany). Further characterization was done with the aid of X-ray Photoelectron Spectroscopy (XPS) analysis, and Fourier Transform Infrared (FTIR) studies.

### 2.4. Analysis of anti-bacterial activity

The normal Agar disc diffusion assay doesn't yield promising results on the anti-bacterial activity of graphene. Hence in the current study we made use of the ‘Colony counting method’.

#### 2.4.1. Growth of bacterial strains

The anti-bacterial assay of exfoliated graphene was performed using the colony counting method on both Gram negative and Gram positive bacteria. Both LB agar (Luria Bertani broth with 1.5% agar) and brain heat infusion agar plates were prepared separately and made to culture with *Escherichia coli* (Gram negative) and *Staphylococcus aureus* (Gram positive) at 37 °C and 30 °C respectively. To 5 mL of broth, a bacterial single colony was inoculated and allowed to culture overnight under standard temperature. Using this 5 mL culture, a total of 50 mL volume of bacteria was harvested. At an optical density (OD-600) of 0.6, each cell was isolated centrifuged at 3500 rpm at a temperature of 4 °C for about 30 min. The remaining excess media components present in the cells were eliminated by washing the isolated cells with saline solution (9% NaCl) twice. These purified cells were then suspended in the same saline solution followed by the quantitative analysis of cells using the spectrophotometer.

#### 2.4.2. Assay of antibacterial activity of exfoliated graphene

Serial dilution was performed before incubating the micro-organisms to the agar plates. Varying concentrations of (0.5, 1 and 1.5 μg ml^−1^) exfoliated graphene solutions were prepared in saline, and population concentrations of 10^7^ cells per ml were incubated in it at 200 rpm for 3 h under room temperature. These incubated bacteria were then serially diluted and finally made to a volume of 10^4^ cells per ml. From this serially diluted sample, 100 μl (containing the progenitor) was spread on the prepared agar plate followed by overnight incubation. The aggregates of cells formed on the plate which were derived from the original progenitor are called ‘colony-forming unit’ (CFU) and were counted to determine the antibacterial activity of the exfoliated graphene. This same scheme was done using distilled water instead of graphene as the control group. The loss of viable cells in the colony on each plate gives the anti-bacterial effect of exfoliated graphene. The number of CFU in the experimental group to that of the control group will give the total viable cell loss in terms of percentage. The whole experiment was repeated thrice and average values were taken for analysis.

### 2.5. Analysis of anti-cancerous activity

The cytotoxicity of biosynthesized graphene was assessed by studying its effects on Dalton's Lymphoma Ascites cells (DLA cells). The cancer cells were obtained from the peritoneal cavity of tumor-carrying mice and subsequently washed three times with Phosphate Buffered Saline (PBS). The main focus of this study is to ascertain cell viability, which was achieved by employing the trypan blue exclusion technique. Tubes containing different concentrations of exfoliated graphene (10 μg, 20 μg, 30 μg, 40 μg, and 50 μg) were prepared, and each tube was combined with a viable cell solution containing 1 × 10^6^ cells in 0.1 ml. To achieve a total volume of 1 ml, phosphate buffer solution was added to each tube, except for the control tube which contained only the cell suspension. The resulting mixture was then incubated for over three hours at 37 °C. Subsequently, the cell solution was mixed with 0.1 ml of 1% trypan blue, allowed to settle for 2–3 minutes, and then transferred to a hemocytometer. The dead cells absorbed the blue tint of trypan blue, while the living cells remained unstained. The total quantities of stained cells along with the unstained cells were then determined by direct counting.^32^
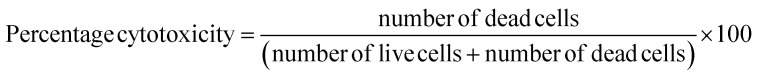


## 3. Results and discussion

A completely new and easily accessible biological approach was employed in the present study for the exfoliation of few-layer graphene (FLG) using the defensive secretion of the beetle *L. tristis*. The graphene sheets produced by an eco-friendly approach in the current study displayed biochemical properties^5^ such as anti-cancerous and anti-bacterial activity. The biological approaches for graphene exfoliation are becoming more and more significant nowadays due to their eco-friendly nature. For the last two decades, various biological agents have been using for the same purpose. But most of these methods are based on plant origin and only a few are of animal origin.^25–27^ To our knowledge, no prior research has been done on the exfoliation of few-layer graphene (FLG) using the defensive secretion of a beetle.

### 3.1. Exfoliation of graphite dispersion using the defensive secretion of the beetle *Luprops tristis*

**Fig. 2:**
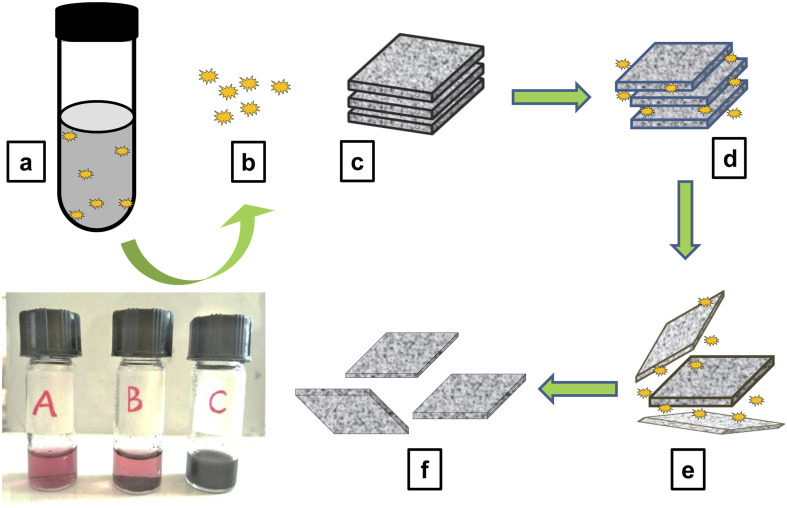
(A–C); (A) red coloured defensive extract of 60 beetles, (B) mixture of defensive gland extract and graphite solution (immiscible) before sonication and (C) the black dispersion formed after sonicating for 1 hour containing the exfoliated graphene. In the diagram: (a) reaction mixture containing the defensive secretion of 60 beetle and the graphite solution in deionised water. (b) The polyphenolic compounds present in the defensive secretion of the Mupli beetle. (c) Stalked graphene layers which constitute the bulk graphite (present in the graphite solution). (d) Adsorption of graphene layers on the surface & edges of graphite and weakening of the van der Waals forces. (e) Intercalation of polyphenolic compounds in between the graphene layers. (f) Separation of graphene layers.

The defensive secretion of the beetle *L. tristis* together with the dispersion of graphite in deionised water was used for the exfoliation graphite dispersion. Graphene were exfoliated efficiently in the form of layers using this method. On the addition of the graphite dispersion (10 μg/400 μl), the deep red coloured solution of gland extract turned in to an immiscible black mixture (Fig. 2 A–C). After ultrasonication for 1 h, the sample developed a distinct black dispersion. This colour change from deep red to the black and the formation of the dispersion indicated the exfoliation of graphite in to graphene.

In the field of graphene synthesis, our research aligns with earlier eco-friendly methods. Chabot *et al.* (2013) pioneered this approach by using Gum Arabic for graphite exfoliation in water, which resulted in graphene with fewer defects and higher conductivity compared to reduced graphene oxide.^34^ Similarly, G. George *et al.* (2018) employed natural polyphenols like curcumin, producing few-layer graphene with minimal defects.^35^ Complementing these efforts, Salunke and Kim, along with Ahadian and colleagues, utilized plant extracts and bovine serum albumin, respectively, for graphene dispersion.^24,25^ These studies collectively underscore a significant shift toward sustainable practices in graphene synthesis, emphasizing the use of green materials and methods. This trend not only supports the development of high-quality graphene but also reflects a commitment to environmentally responsible scientific practices, addressing the needs of the future.

There are only a few studies about the Mupli beetle, their morphology, life history,^29^ population dynamics,^30^ biological control,^28^ diapause period^36^ and structure of defensive gland.^37^ Our study reveals that the defensive secretion of the mupli beetle has the capacity to exfoliate graphene from a sample of graphite dispersion. Like in the case of electrochemical liquid phase exfoliations, the defensive secretion can play the role as a potent dispersant here. Furthermore, the glandular extract also acts as a good stabilizing agent. GC-HRMS analysis was recently used to determine the chemical makeup of the defensive glandular extract of the Mupli beetle, *L. tristis*.^32^ It disclosed the presence of polyphenolic compounds and pheromones such as 1,3-dihydroxy-2-methylbenzene, 2,3-dimethyl-1,4-benzoquinone, 2,5-dimethyl hydroquinone, oleic acid, pentacosane, tetracosane, hexacosane, *tert*-hexadecanethiol and 7-hexadecenal. Furthermore, this study also disclosed the antioxidant, antimitotic, cytotoxic and antibacterial properties of the defensive gland of the mupli beetle and thereby bringing profitable biological activities of the defensive gland to the limelight. A former study on different species of the Tenebrionid beetle by Brown and co-workers had pointed out that the main chemical constituent in the defensive secretion of this family may be quinones.^38^ Since polyphenolic compounds have good reducing, capping and anti-oxidant strengths, they provide stability. The phenolic compounds present in the gland extract due to their distinct diffusability and minute size get adsorbed on the graphite surface. The energy gained through the sonication results in the intercalation of these compounds in between the graphite layers (Fig. 2a–f). As the pressure develops the weak Van der Waals force which holds together the sp^2^ carbon atoms get weakened, and the spacing of graphite layers increase from 0.34 nm to a higher value. The continuously acting sonication energy and the intercalation together with the release of reactive oxygen species (ROS) by the phenolic compounds may be leads to the exfoliation of graphite into few-layer graphene.^39,40^ Recent studies by Song and co-workers (2020) showed that there are three separate phases in the conversion of graphite flakes to graphene. The initial phase of sonication leads to the fragmentation of large flakes and the appearance of kink band striations, which are notably observed in zig-zag patterns on the surfaces of the flakes. In the subsequent phase, fissures form along these striations, and when the solvent is intercalated, small graphite strips begin to unzip and peel off, ultimately resulting in the exfoliation of graphite.^41,42^

Ultra-sonication techniques have significantly improved the conventional methods of producing graphene using different solvents, which were previously tentative and yielded minimal results. The introduction of ultra-sonication has facilitated the creation of nanoparticles with excellent stability, making the process more efficient.^43^ Tyurnina and colleagues conducted a study demonstrating the feasibility of rapidly and precisely controlling the generation and quality of few-layer graphene flakes in pure water by investigating the impact of critical ultrasonic liquid phase exfoliation (LPE) parameters.^44^ Similarly, another study found that employing basic moderate bath sonication at higher frequencies and lower power levels enhances the size, thickness, and quality of the resulting exfoliated particles.^45^ More in-depth studies are required to confirm the various phases associated with the formation of few layer graphene using the defensive extract of the *L. tristis*. A further thorough investigation is required to confirm the various steps involved in synthesizing a few layer graphene using the defensive extract from *L. tristis*.

### 3.2. Characterization of exfoliated graphene

The exfoliated graphene was characterized using various techniques, including Transmission Electron Microscopy (TEM), Atomic Force Microscopy (AFM), Raman Spectroscopy, X-ray Photoelectron Spectroscopy (XPS) analysis, and Fourier Transform Infrared (FTIR) studies. The obtained results are as follows.

#### 3.2.1. Transmission electron microscopy (TEM)

**Fig. 3:**
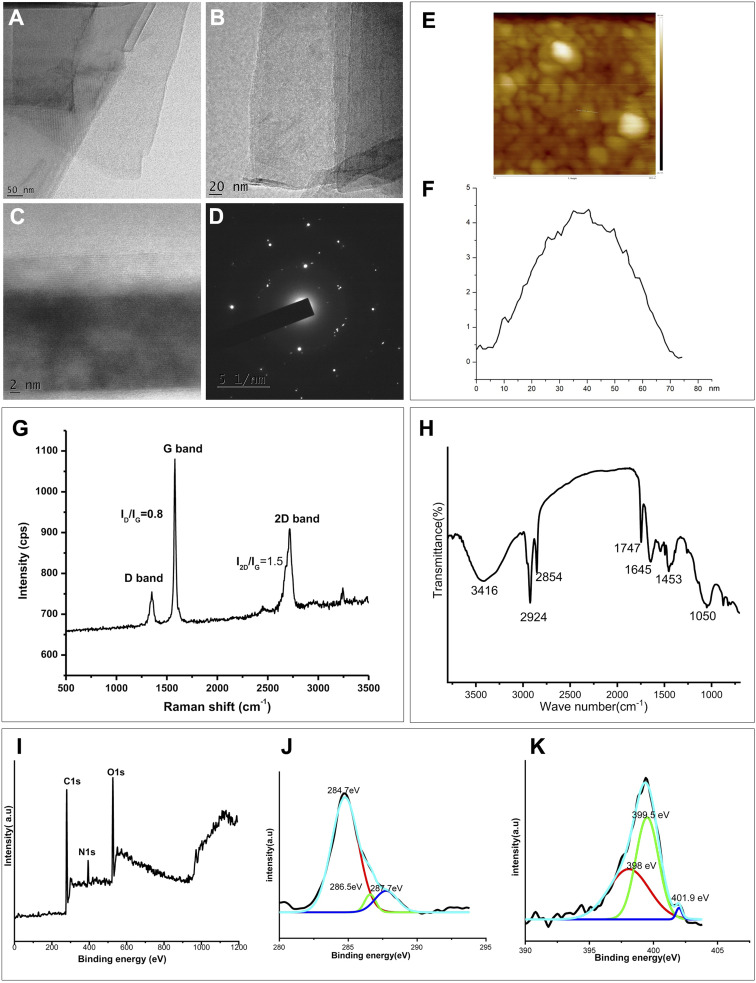
(A–C) TEM images of exfoliated graphene layers with dimensions of 50 nm, 20 nm, and 2 nm respectively, (D) electron diffraction pattern (EDP) of the exfoliated graphene layer (51 nm), (E) AFM profile of the exfoliated graphene. (F) Height of the exfoliated graphene obtained from AFM analysis, (G) Raman spectra of the exfoliated graphene. (H) FTIR data of N-doped graphene, (I–K) PS Spectra of N-doped graphene; (I) survey spectrum (J) C 1s deconvoluted spectrum and (K) N 1s deconvoluted spectrum of N-doped graphene.

The morphology and number of layers of the prepared graphene can be characterized using transmission electron microscopy.^3^ The TEM images of the sample obtained are given in figures (Fig. 3A–C) which demonstrate good degree of exfoliation. These representative images show a wrinkled morphology with few stalked layers at the edge. TEM images show the presence of few-layer graphene (FLG) in the sample.^46^ The selected area electron diffraction pattern of a particular layer of the exfoliated graphene is given (Fig. 3(D)). This picture shows a diffraction pattern oriented in 6 edges, representing the hexagonal structure of the graphene. The light emitted in this hexagonal symmetry confirms that the distortions are altogether absent in the product during the exfoliation process.^47^ The TEM examination indicates that the exfoliation method, employing beetle extract, effectively yielded FLG.

#### 3.2.2. Atomic force microscopy (AFM)

AFM is a very trustworthy tool in nanotechnology for examining the thickness, surface structure, and chemistry of the nanoparticles. The recent research by Kumar *et al.*, (2021) provides a thorough overview of the AFM analysis used to gauge graphene thickness. Graphene thickness has been quantified through the vertical and lateral dimensions acquired from AFM examination.^48^ The AFM image of the exfoliated graphene is presented in Fig. 3(E and F). Using the AFM height profile of the graphene, 2.163 nm was determined to be the thickness of exfoliated graphene. A single sheet of graphene being approximately 0.345 nm thick, this proves that the exfoliated sample contains FLG, which is normally found in layers between 5–8.^25^

#### 3.2.3. Raman spectroscopy

The Raman spectrum of the exfoliated graphene is given in figure (Fig. 3G). A Raman spectrum helps to examine the sample quality and number of layers present in the sample.^49^ In addition, it also provides information on the structure and chemical properties of our desired sample.^49^ The Raman spectrum of the exfoliated graphene shows mainly 3 peaks^50^ which can be assigned as D band, G band 2D band seen around 1340, 1580 and 2700 cm^−1^ respectively. A distinct, sharp G band can be observed in the spectrum, which indicates the graphitic nature.^6^ As the G band is sharper, it reflects the highly crystalline nature of the graphene formed.^51,52^ The presence of G band is due to the in-plane vibration. The position of G band tells about the number of layers of graphene formed and its intensity reflects the thickness of layers.^53^ The weak D band indicates the low defects in the graphene layers.^54^ The G and D band confirm the formation of highly crystalline, defect-less graphene sheets. Presence of strong 2D band is a characteristic of graphene which indicates the 2nd order defects and thickness of the layers.^49,55^ The *I*_D_/*I*_G_ ratio gives the density of defects. Here the *I*_D_/*I*_G_ ratio is found to be 0.8 which indicates that the graphene formed is of fewer defects. The *I*_2D_/*I*_G_ ratio of 1.5 for the graphene exfoliated with beetle defensive extract indicates that the sample is composed of few layers. This conclusion is derived from the general fact that an *I*_2D_/*I*_G_ ratio above 2 is characteristic of monolayer graphene, while a ratio below 2 suggests the presence of more than one layer in the exfoliated graphene. Consequently, the observed *I*_2D_/*I*_G_ ratio of 1.5 aligns with the properties of few-layer graphene.^48^ So the Raman spectrum confirms the formation of a ‘Few Layer Graphene (FLG)’ in our sample.

#### 3.2.4. FTIR spectroscopy

FTIR spectroscopy is a widely used analytical technique and furnishes comprehensive insights into the functional groups in the sample.^56,57^ FTIR analysis of the N-doped graphene is presented in Fig. 3(H) with vibrational frequencies at 1050 cm^−1^ (C–O stretching vibration), 1453 cm^−1^ (O–H bending), intense bands at 1645 cm^−1^ (C=C and C=N vibrations), 1747 cm^−1^ (C=O), 2924 and 2854 cm^−1^ (C–H stretching), 3416 cm^−1^ (O–H stretching). This signifies the influence of polyphenolic compounds from the defensive extract^32^ that facilitate surface functionalization, thereby contributing to facile exfoliation of graphite into graphene *via* sonication. The nitrogen containing molecules in the defensive gland extract of the beetle aid in elemental doping of N into graphene.

#### 3.2.5. X-ray photoelectron spectroscopy (XPS)

X-ray Photoelectron Spectroscopy is a powerful tool to analyse the electronic configuration and composition of the elements in the fabricated nanomaterial. The derived binding energy values obtained from the XPS analysis render information on the nature of binding between elements present in the sample.^58^Fig. 3(I) shows the survey spectrum of as prepared sample with peaks of C 1s, N 1s and O 1s.The atomic percentage of C, N and O obtained from the XPS analysis are 68.84%, 7.81% and 23.35% respectively. Fig. 3(J) depicts the high resolution deconvoluted spectra of C 1s with three distinct peaks. The prominent peak at 284.7 e V corresponds to graphitic C=C species, the peak at 286.5 eV corresponds to the sp^3^ carbons (C–OH) and the binding energy at 287.7 eV may corresponds to C=O or C–N.^59–61^

High resolution N 1s spectrum, shown in Fig. 3(K) reveals the binding configuration of nitrogen atom in the graphene sample. The presence of different N atoms in the sample is apparent through the observation of three spectral peaks with binding energy values at 398 eV (for pyridinic N), 399.5 eV (for pyrrolic N) and 401.9 eV (for graphitic N).^62^ The significance of N-doped graphene is that N-doping might introduce a change in the Fermi level and may result in the graphene band gap opening. Thus the presence of oxygen functionality (which impart to the stable aqueous dispersion) as well as the nitrogen doping in the sample is confirmed from FTIR and XPS analysis.

### 3.3. Antimicrobial activity of the exfoliated graphene

**Fig. 4:**
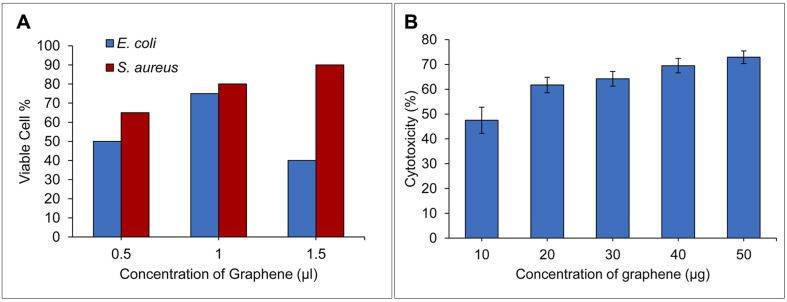
(A) Bar diagram showing the anti-bacterial assay of exfoliated graphene on both Gram positive & negative bacteria, (B) bar diagram showing the cytotoxicity of exfoliated graphene on DLA cells.

The anti-bacterial activity of the exfoliated graphene using the defensive secretion of the beetle *L. tristis* was tested using the colony counting method as shown in Fig. 4(A). Exfoliated graphene displayed notable antibacterial properties against both the Gram negative and Gram positive bacteria. A maximum of antibacterial activity was observed at the concentration of 1.5 μg ml^−1^ for *S. aureus* and 1.0 μg ml^−1^ for *E. coli.* The bacterial colonies on the plate treated with exfoliated graphene solution showed an intensive anti-bacterial activity than the control group. The main reason behind the antibacterial properties of graphene is believed to be its physicochemical interaction with bacteria.^63^ Recent findings have proposed three primary mechanisms through which graphene sheets act as antibacterial agents: (1) nano-knives: the sharp edges of graphene cause damage to bacterial cells. (2) Wrapping or entrapment: graphene's dynamic thin film structure can wrap around or entrap bacterial membranes, leading to their disruption. (3) Oxidative stress: graphene can induce oxidative stress in bacteria, either with or without the generation of reactive oxygen species (ROS).^64–69^

In the current investigation, bacteria may got trapped between the thin graphene layers, the highly lipophilic edge and surface of the exfoliated graphene may disrupt the membrane easily^70^ together with the direct DNA damage by the reactive oxygen species (ROS) released by the polyphenolic compounds of the defensive gland extract may be the possible reason for the ultimate death of bacterial colonies. The exfoliated graphene showed a stronger bactericidal effect on *S. aureus* than the *E. coli*. It can also be noticed Gram-negative (*E. coli*) and Gram-positive bacteria (*S*. *aureus*) have different bacterial cell membranes, which may account for the variation in their antibacterial properties. Graphene nanomaterial can easily penetrate and damage the peptidoglycan layer of Gram-positive bacteria because they lack the extra lipophilic membrane that is present in Gram-negative bacteria and provides them with additional strength and protection.

### 3.4. Anti-cancerous activity of exfoliated graphene

The cytotoxicity analysis showed biosynthesized graphene showed *in vitro* anticancer activity against DLA cells Fig. 4(B). The high concentration of biosynthesized graphene (10–50 g ml^−1^) has been found to gradually strengthen the cytotoxicity. The various physicochemical characteristics of graphene sheets, their framework, size, composition and the raw material utilised to produce graphene may eventually affect how they interact with cells, which will also determine their and its cytotoxicity.^15^ The observed cytotoxicity in the present investigation could be attributed to the combined effects of graphene's inherent physical characteristics and the polyphenolic compounds from the defensive glands, which are appended to the graphene surface during the exfoliation process. The interaction of these elements with DLA cells may be responsible for inducing cell death. According to research by Liao and colleagues, the oxides, strong acids, and other organic compounds found in the organic solvents used for exfoliation may become poisonous and may spread their harmful effects to the tumour cells through the contact of the exfoliated product and the tumour cell.^71^ Endocytosis forms the gate through which the novel graphene nanosheets get access to the interior of cell.^72^ In our previous research, we found that the polyphenolic components present in the crude defensive extract of *L. tristis* were toxic to DLA cells.^32^ A comparison between our earlier studies using this extract and the current work with exfoliated graphene reveals that, while both demonstrate an increase in cytotoxicity proportional to concentration, the crude defensive extract is notably more potent in killing DLA cells across both low and high concentrations. The reduction in cytotoxicity of exfoliated graphene may be due to the dilution of polyphenolic compounds during the exfoliation process.

## 4. Conclusion

The study introduces a green method for producing few-layer graphene (FLG) *via* liquid phase exfoliation, employing the defensive secretion from the beetle *Luprops tristis*. It explores the potential of utilizing the extract from the beetle's defensive gland in the exfoliation of graphite to FLG, aided by ultrasound. Furthermore, the exfoliated graphene demonstrated potent antibacterial activity against both Gram-positive (*Staphylococcus aureus*) and Gram-negative (*Escherichia coli*) bacteria. Additionally, the cytotoxic assay indicated the anti-cancerous properties of the exfoliated graphene against Daltons Lymphoma Acetic (DLA) cells. The research also highlights the potential for scaling up the synthesis of few-layer graphene using the synthetic chemicals found in the beetle defensive gland extract. The utilization of the beetle's defensive gland extract as an exfoliation agent presents a significant advancement in the field of graphene synthesis and opens up exciting prospects for various applications in biomedicine and materials science.

## Data availability

The data that support the findings of this study are available upon request from the corresponding author.

## Author Contributions

A. P. A.—supervision, conceptualization, data curation, investigation, review and editing of the original draft. K. N.—investigation, experimental work, and data analysis. O. S. investigation, experimental work, and data analysis K. N. J.—conceptualization, data curation, investigation, review, and editing of the original draft. S. R. V.—formal analysis, review, and editing, A. R. V.—experimental work, data collection, V. S. B.—formal analysis, review, and editing. K. S.—data analysis and interpretation, R. M. R.—data curation review and editing of the data, A. B.—investigation, experimental work.

## Conflicts of interest

The authors declare that they have no known competing financial interests or personal relationships that could have appeared to influence the work reported in this paper.

## Supplementary Material

RA-014-D3RA08733F-s001
