# Supplementary figures and images for: A bio-inspired approach for the synthesis of few-layer graphene using beetle defensive gland extract
Source: RSC Adv. 2024 Feb 16;14(9):5729–39. doi: 10.1039/d3ra08733f (PMC10870200; doi:10.1039/d3ra08733f)

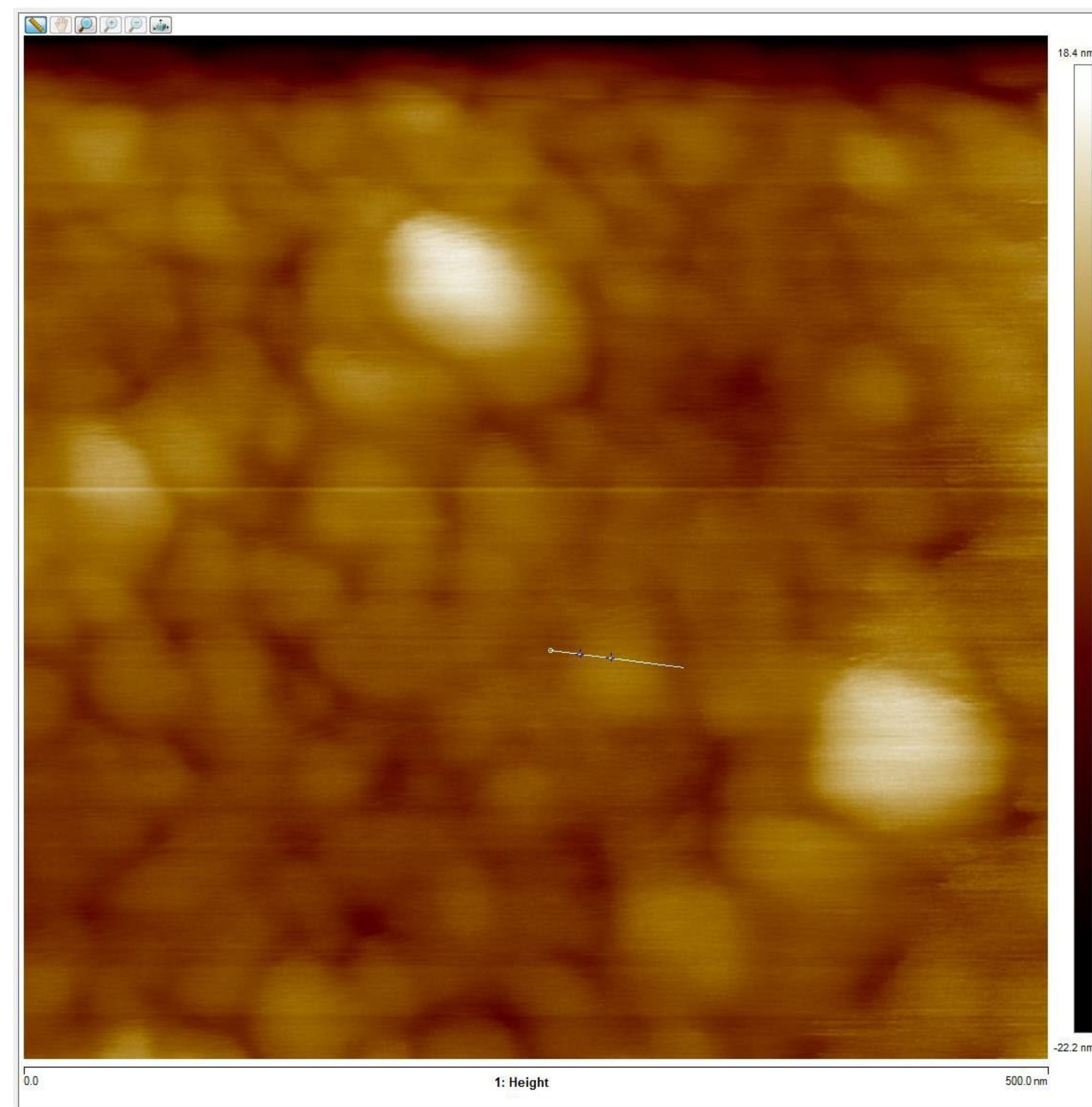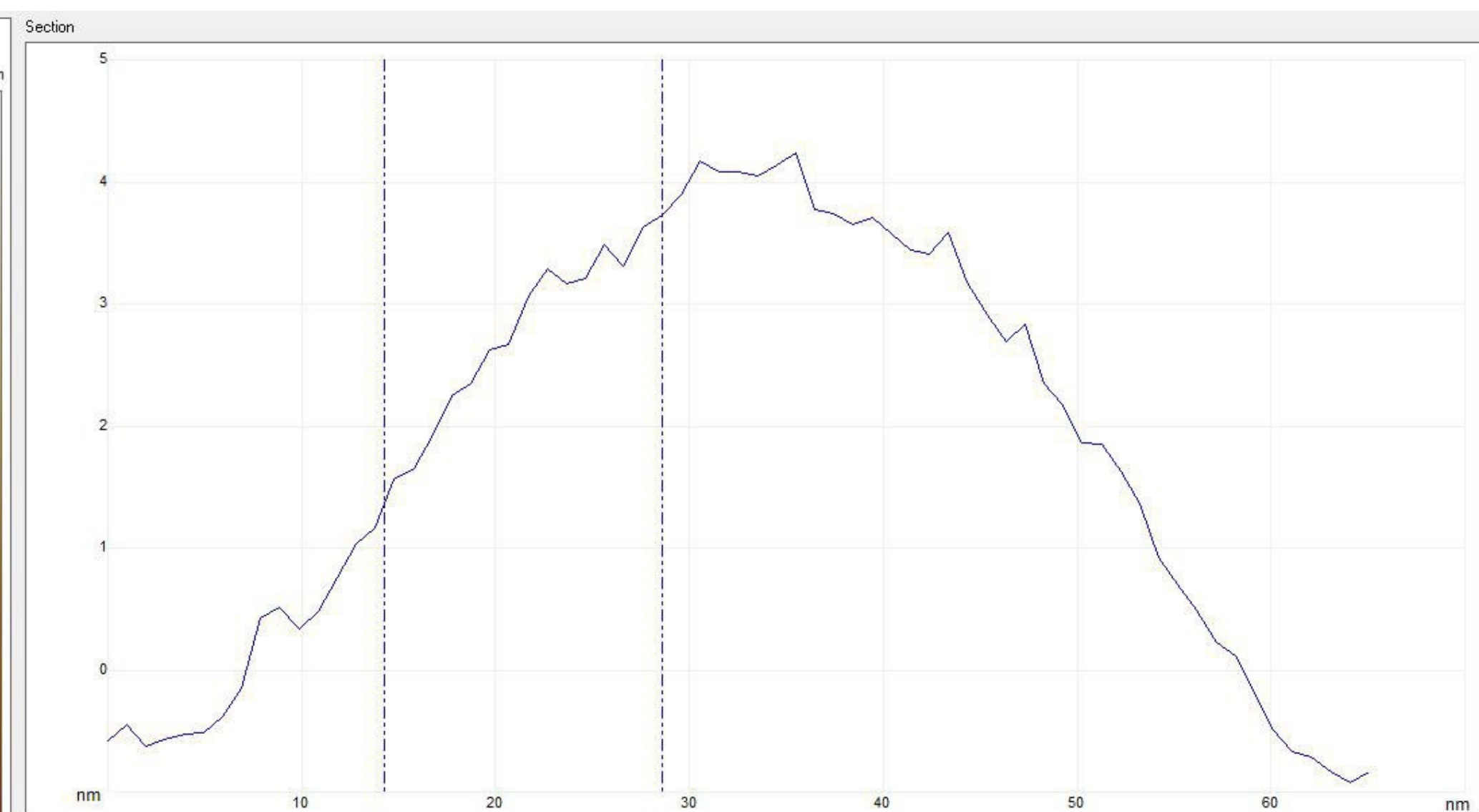

Spectral Period 0.00 nm  
Spectral RMS Amplitude 2.52 nm  
Spectral Frequency 0.00 /nm  
Temporal Freq: 0.00 Hz

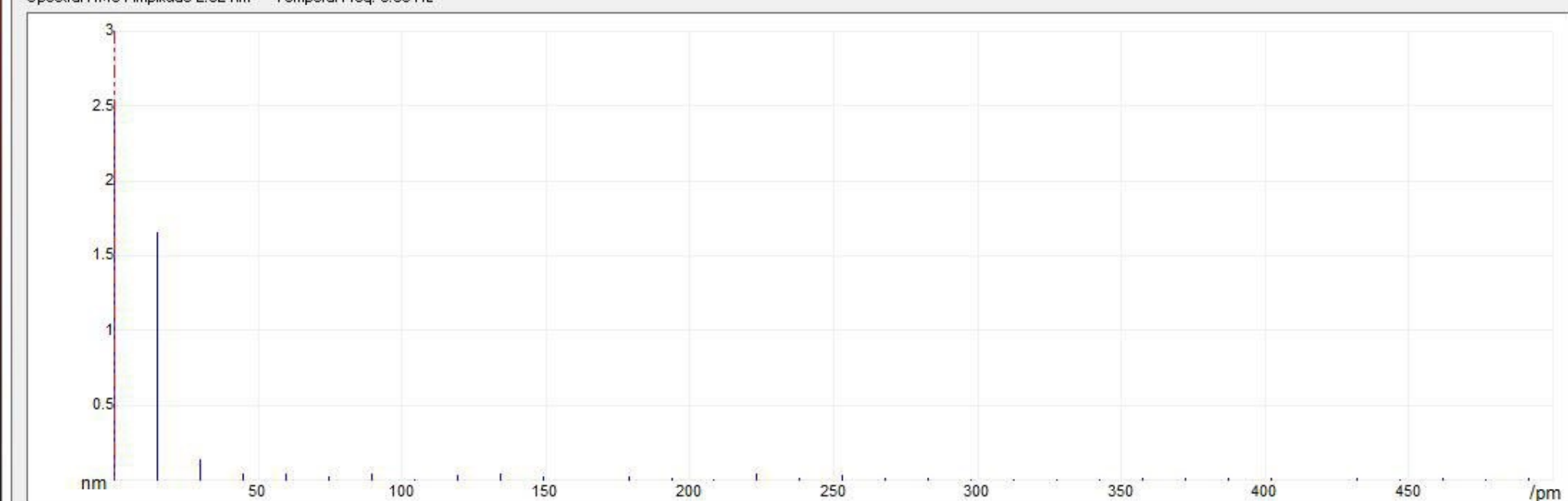

Supplement: RA-014-D3RA08733F-s001 [file RA-014-D3RA08733F-s001.pdf]
